# Response shift masks the treatment impact on patient reported outcomes (PROs): the example of individual quality of life in edentulous patients

**DOI:** 10.1186/1477-7525-3-55

**Published:** 2005-09-07

**Authors:** Lena Ring, Stefan Höfer, Frank Heuston, David Harris, Ciaran A O'Boyle

**Affiliations:** 1 Department of Psychology, Royal College of Surgeons in Ireland, Mercer Street Lower, Dublin 2, Ireland; 2 Dept of Pharmacy, BMC, Box 580, 751 23 Uppsala, Sweden; 3 Department of Medical Psychology and Psychotherapy, Medical University Innsbruck, Innsbruck, Austria; 4 Department of Restorative Dentistry & Periodontology, Dublin Dental Hospital, Dublin 2, Ireland; 5 The School of Dental Sciences, Trinity College, Dublin 2, Ireland

## Abstract

**Background:**

Quality of life (QoL) is now established as an important outcome for evaluating the impact of disease, and for assessing the efficacy of treatments. However, individuals change with time and the basis on which they make a QoL judgement may also change, a phenomenon increasingly referred to as response shift. Here, the individual may change his or her internal standards, values, and/or conceptualization on the target construct as a result of external factors such as a treatment or a change in health status. This has important implications for assessing the effects of treatments as a change in QoL may reflect a response shift, a treatment effect, or a complex combination of both. In this study, we used an individualised quality of life (IQoL) measure, the SEIQoL, together with a then-test to determine whether response shift would influence the measurement of treatment efficacy in edentulous patients.

**Methods:**

Data are reported here for the first phase of a randomised controlled clinical trial designed to assess the impact, on IQoL, of implant supported dentures compared with high quality conventional dentures. IQoL was measured using the SEIQoL-DW in 117 patients (mean age 64.8; 32% male) at baseline (T_1_) and 3 months (T_2_) after receiving high quality conventional dentures. The work was carried out in dental teaching hospitals in Dublin and Belfast.

**Results:**

Unadjusted SEIQoL index scores revealed no significant impact of treatment at three months (baseline: 75.0; 3 months: 73.2, p = .33, n.s.). However, the then-test at 3 months revealed that patients retrospectively rated their baseline IQoL as significantly lower (P < .001) than they had rated it at the time (then-test baseline: 69.2). Comparison of the 3 month scores with this readjusted baseline indicated a significant treatment effect (then-test baseline: 69.2; 3 months: 73.2, p = 0.016). 81% of patients nominated at least one different IQoL domain at 3 months.

**Conclusion:**

The positive impact of denture treatment for edentulous patients on IQoL was seen only when response shifts were taken into consideration. The nature of the response shifts was highly complex but the data indicated a degree of re-conceptualisation and reprioritisation. Assessment of the impact of treatments using patient-generated reports must take account of the adaptive nature of patients.

## 

"I know who I was when I got up this morning, but I've changed several times since then." (Alice in Wonderland by Lewis Carroll)

## Background

Patient reported outcomes (PROs) are widely used in clinical trials to incorporate patients' perspectives, and as adjuncts to "harder" measures of morbidity and mortality [1]. While PROs have long been considered simple, valid and reliable measures of outcome, it is increasingly clear that the cognitive processes involved in completing them are complex [2]. Recently attention has been drawn to potentially highly significant phenomena known as response shift [3]. As human beings, we actively construct meaning from our environment, and display a range of cognitive mechanisms to continually adapt to changing circumstances. Response shift refers to a change in the meaning of one's evaluation a construct as a result of a change in one's internal standards of measurement, a change in one's values, or a change in one's definition of the construct [3]. This means that persons might give different answers on PRO measures over time, not only because their health or quality of life (QoL) has changed, but also because they might have changed their perception on what health or QoL means to them. This may be particularly important in repeated measures trials where efficacy is measured as the change from a pre-treatment baseline following treatment.

QoL is highly individual with patients varying considerably in what they consider important for their QoL. It is also know that the elements of QoL change over time and in response to changing circumstances [4]. Individualised measures of QoL (IQoL) increase respondents' discretion in selecting the domains most important to their QoL and, in determining the relative importance of these domains. While it is feasible to use IQoL measures [4], they are not yet widely used in clinical trials [5]. IQoL measures such as the Patient Generated Index (PGI) [6] and the Schedule for Evaluation of Individual Quality of Life (SEIQoL) [7] may prove useful in determining the impact of response shift on the assessment of treatment effects in clinical trials.

The present study was designed to evaluate response shift in edentulous patients undergoing treatment. This is a chronic dental condition defined as total tooth loss and which can have a significant impact on QoL. Patients seek treatment for aesthetic reasons and to restore their oral function. Whereas biological outcome measures such as pocket depth, bone loss and chewing ability are important in this patient group, there is an increasing emphasis on the use of satisfaction and QoL measures to assess the impact of treatment [8]. Previous research has shown low correlations between patients' evaluations of their prostheses and clinicians' biological assessments [9].

Modern approaches to treatment involve replacing conventional dentures with dentures supported by osseointegrated implants set into the bone. Improvements in oral health following implants are well documented [10] and implant-supported dentures are considered superior to conventional dentures, since they are experienced as a part of the patient's own body and allow patients to feel food textures. Implants are more expensive than conventional dentures and the treatment is also somewhat more cumbersome since it involves dental surgery to attach the implants. Few longitudinal studies have used QoL as an outcome to compare implants and conventional dentures [8]. This study was designed as a randomised clinical trial comparing treatment with implants with high quality conventional dentures with QoL as the main outcome measure. Data are reported here only for the first phase of the study in which all patients wore new high quality (best possible) conventional dentures for three months before being randomised either to continue with the conventional dentures or to receive implants. The fitting of excellent dentures was done to ensure a proper baseline for the clinical trial since many edentulous patients report problems with ill-fitting dentures. This is the first longitudinal study to use the IQoL measure SEIQoL-DW [11] in this patient group.

We hypothesised that fitting high quality dentures or osseointegrated implants was likely to result in improved eating, communication, appearance and social life [12] and that consequently there should be a significant improvement in individual IQoL. Since the treatment periods were long (three months and six months), the psychological impact of treatment was likely to be marked on the main outcome measure individualised QoL. Therefore we decided to attempt to measure response shifts, since should these occur, they would complicate the interpretation of the IQoL data.

## Methods

Data presented here were collected as part of a larger randomised controlled clinical trial comparing conventional dentures with osseointegrated implant supported dentures in edentulous patients. The Ethics committees of the respective dental teaching hospitals in Dublin and Belfast granted ethical approval. Eligible patients were recruited from waiting lists and from dental practices. Patients, identified by consecutive sampling, were invited to participate until the planned sample size of 70 patients per centre was reached. Patients had to have been edentulous for at least two years, be under 75 years of age, be medically suitable for surgery, be either non-smokers or smoking fewer than 10 cigarettes per day, have bone height at the anterior mandible on radiographic assessment of at least 1 cm, have the cognitive ability to understand the purpose of the study, and they had to provide informed consent. All patients were fitted with the highest possible quality conventional dentures at baseline and they wore these for three months before being randomly assigned either to continue with the conventional dentures or to receive implant supported dentures. This was designed to establish a clinically acceptable baseline treatment to assess the added value, if any, of implant supported dentures.

### Individualised quality of life (IQoL)

IQoL was measured using the Schedule for the Evaluation of Individual Quality of Life – Direct Weighting (SEIQoL-DW) [11]. Respondents were first asked to nominate and describe the 5 areas of their lives (cues) that they consider to be the most important for their QOL. They were then asked to rate their current level of satisfaction/functioning on each cue on a scale between worst possible and best possible. Finally, they were requested to allocate 100 points to indicate the relative importance of each cue by using a pie-chart disc. The SEIQOL Index summary score, was derived by multiplying each cue's weight by its corresponding level, and summing the products across the 5 cues. The SEIQOL Index score ranged from 0 – 100, where a higher score indicates better QoL.

### Response shift

The SEIQOL allows patients to nominate different cues at each assessment. It was assumed that, patients who nominated different cues as being important to their QoL at 3 months has changed their concept of what constituted QoL for them. This may reflect what Schwarz and Sprangers refer to as re-conceptualization (Table 1).

**Table 1:** SEIQoL-DW components of response shift

**Response shift**	**Description**	**SEIQoL-DW indication**
Re-conceptualisation	Change in one's definition of the target construct	Changes in nomination of cues (life areas) when comparing pre- and post-test cue nominations.
Re-calibration	Change in one's internal standards of measurement	Changes in cue (life area) levels when comparing the pre-test and then-test scores
		Changes in SEIQoL Index scores when comparing pre-test and then-test scores
Re-prioritisation	Change in one's values	Change in cue (life area) weights when comparing the pre- and then-test scores

In addition to assessing re-conceptualization in the form of different cue nominations, we also wanted to determine whether other types of response shift might occur which could impact on the assessment of the treatment. Treatment effects are usually determined by assessing changes in scores from a pre-treatment baseline. It is assumed that a change represents a treatment effect. However, if a respondent shows a response shift by changing the manner in which he or she completes the measure, this may confound interpretation of the treatment effect. The then-test has been proposed as a method that allows some aspects of this process to be assessed [13]. The patient is asked at T_2 _retrospectively to rate their situation at T_1_, not as they recall it, but as they now see it. The theory is that they should be using the same internal criteria for rating T_1 _as they are now using for rating T_2_. Differences between the original T_1 _rating and the retrospective T_1 _rating indicate response shift. If such differences are found, the difference between the retrospective T_1 _score and the T_2 _score is a more accurate indication of the treatment effect since it controls for the response shift. For example, a patient rates her pre-treatment level of pain as 7 on a 10-point pain scale. She subsequently rates her post-treatment level of pain as 3. This is taken to indicate that the treatment has caused an improvement of 4 points. However, if she retrospectively rates her pre-treatment pain as having been a 5, the actual treatment effect is 2. Likewise, if she retrospectively rates her pre-treatment pain as having been 10, the actual treatment effect is 7. The theory proposes that she is using the same internal standard to make the retrospective assessment as she is using to make the current assessment. It can be seen from this example that a response shift in a PRO may cause one to overestimate or underestimate the real effect of a treatment if one is basing the judgement solely on changes in raw scores.

We used the then-test at three months by asking patients to rate retrospectively their original cues at baseline. They were asked to re-rate cue levels (*re-calibration*) and cue weights (*re-prioritisation*). The hypothesised components of response shift measured in this way are shown in Table 1. The then-test was administered using the following wording:

"You have shown me how your quality of life is at the moment. The five important life areas that you have spoken about today are the same five areas that you chose when we first met^1^. I would like you to show me how you now think you were doing in each of these five life areas when we first met, by using this same scale that we used earlier. ...I am not asking you to try and remember how these important life areas were functioning, but rather how, when looking back today, you now think they were functioning when we first met."

Where patients had selected different cues at T_2 _they were asked in the then-test to retrospectively rate the cues originally chosen at T_1_.

The following wording were used to assess re-prioritisation:

"Now I would like you to show me how important you now think your five life areas were in relation to each other when we first met. Once again, I am not asking you to try and remember how important these life areas were in relation to each other, but rather what, when looking back today, you now think was their relative importance when we first met."

Three experienced dental nurses, trained in the SEIQoL interview technique and using a standard protocol for administration of the then-test, administered all assessments.

### Statistics

Data were described using frequency distributions. Paired t-tests were used to assess changes from T_1 _to T_2_. Differences at a specific time point between variables were assessed by independent t-tests. P values of p < .05 were considered significant.

## Results

Complete data were available for 117 patients (83.6%) at baseline (T_1_) and 3 months after (T_2_) receiving high quality conventional dentures. The mean age of the sample was 64 ± 8 years and 32% were male. The aim was to include 140 patients but the final sample consisted of 136 patients. Two patients died before follow-up, others dropped out due to having cancer (n = 1), or due to having sick or dying relatives (n = 2), or were withdrawn due to denture/implant problems (n = 4) and some dropped out without reason (n = 5). Of the 122 patients finally included at T1, 117 completed all study measures at both time points. None of the patients found it difficult to complete SEIQoL.

### Cues nominated

The most frequently (percent of patients, choosing the category as cue 1 and 5) nominated cues were; Family/next of kin (46%–4%) e.g., partner, brother, mother, children, grandchildren; Health (34%–5%) e.g., stay healthy, being fit, being alive, pain; Hobbies/recreation (2%–31%) e.g., sports, reading, dancing, golf; Social life (2%–8%) e.g., meeting up with friends, ill fitting dentures preventing social life, embarrassed to eat and talk; and Religion/faith (3%–11%) e.g., God is important in life, like to believe there's something after life. Cues directly concerned with dental function were less frequently nominated (5%–3%) e.g., oral health, eating out, dentures, being able to eat/talk properly and appearance.

### Treatment effect

**Figure 1:**
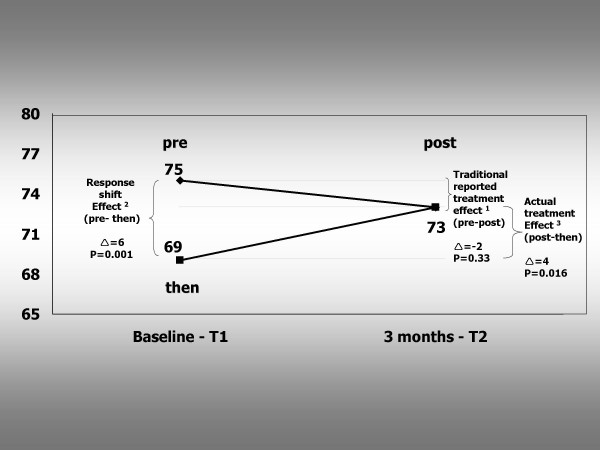
**SEIQoL Index scores at baseline (T_1_) and after three months (T_2_). **^1^The *traditional reported treatment effect *= **post-test **minus **pre-test **score ^2^The *response shift effect *= **pre-test **minus **then-test **score ^3^The *actual treatment effect *= **post-test **minus **then-test **score

As shown in Figure 1, there was no significant change in unadjusted SEIQoL-DW Index scores from baseline to 3 months following treatment (pre-test: 75.0; post-test: 73.2, p = .33). Baseline Index scores generated from retrospective assessments using the then-test were significantly lower that the Index scores generated using the original baseline data (original baseline: 75.0; then-test baseline: 69.2, p < .001). When Index scores at 3 months were compared with the baseline scores generated using the then-test, a significant improvement in IQoL following denture treatment was found (then-test: 69.2; post-test: 73.2, p = 0.016).

### Response shifts

#### Cues

81% of patients nominated at least one different cue at 3 months compared to baseline.

#### Cue levels

In the then-test, all patients were asked to retrospectively rate their baseline cue levels and weights at 3 months even if they had nominated new cues at the latter time. As shown in Table 2, mean cue levels ranged from 76-73 (pre-test), 68–75 (post-test) and 68–70 (then-test) on the scale from 0–100. In the then-test, patients significantly changed their ratings of the cue levels of 4 of 5 cues, on average, indicating a significant degree of re-calibration. There was a significant change from baseline at 3 months in level of the most important cue (the cue given the highest relative weight), when comparing post-test and then-test scores. While the most important cue nominated at 3 months is not necessarily the same as that nominated at baseline, the change in the average level of the most important cue may reflect a significant treatment effect.

**Table 2:** Average cue levels and weights for each of the 5 SEIQOL-DW cues selected by respondents at baseline.

Cues*	T_1 _– pre-test mean level ± SD. mean weight ± SD.	T_2 _– post test mean level ± SD. mean weight ± SD.	T_1 _– then-test mean level ± SD. mean weight ± SD.	P-value Levels #	P-value Weights #
1				pre-post: .359	pre-post: .026
level	73.8 ± 28.3	74.6 ± 22.3	69.7 ± 26.9	pre-then: .076	pre-then: .033
weight	29.6 ± 12.4	26.9 ± 9.2	26.7 ± 9.79	post-then: .005	post-then: .649
2				pre-post: .088	pre-post: .404
level	76.2 ± 24.1	71.1 ± 26.2	69.0 ± 24.7	pre-then : .003	pre-then .152
weight	23.3 ± 10.59	22.3 ± 9.47	21.8 ± 8.61	post-then: .106	post-then: .598
3				pre-post: .069	pre-post: .801
level	75.7 ± 27.1	70.8 ± 24.0	70.1 ± 24.7	pre-then: .006	pre-then: .534
weight	20.2 ± 9.91	20.6 ± 8.85	21.0 ± 8.97	post-then: .403	post-then: .808
4				pre-post: .269	pre-post: .223
level	72.7 ± 24.7	68.1 ± 26.4	67.9 ± 23.8	pre-then: .036	pre-then: .073
weight	15.0 ± 7.91	16.2 ± 8.27	16.6 ± 7.75	post-then:.424	post-then:.576
5				pre- post:.105	pre- post:.012
level	74.2 ± 22.6	69.5 ± 25.7	68.7 ± 23.9	pre-then: .028	pre-then:.015
weight	12.3 ± 6.05	14.2 ± 7.03	14.3 ± 6.50	post-then: .635	post-then: .777

* Cues in descending order of importance at T1

# The *traditional reported treatment effect *= **post-test **minus **pre-test **score

# The *response shift effect *= **pre-test **minus **then-test **score

# The *actual treatment effect *= **post-test **minus **then-test **score

#### Cue weights

Mean cue weights (see Table 2) ranged from 12%–30% (pre-test) and 14%–27% (post-test) and 14%–27% (then-test). In the then-test (comparing pre-test and then-test scores), patients significantly changed their weightings, on average, for the most and least important cues. At 3 months, the weights assigned to the most and least important cues were significantly different than those assigned to the most and least important cues at baseline (Note: the most important and least important cues nominated at 3 months are not necessarily the same as those nominated at baseline).

## Discussion

The main focus of this paper is the significance of response shifts for assessing treatment outcomes. Disparities between objective clinical measures and patients' subjective assessments are common. Patients with the same condition respond differently and even the same patient can respond differently over time. QoL measures used currently in clinical research were not designed to account for response shifts but are based on the assumption that people respond consistently on measurement scales and also that scales are directly comparable across individuals and over time. The classical approach has been to consider individual differences in response as sources of error. However, Schwartz and Rapkin [14] have argued convincingly that individual differences in cognitive appraisal processes should be viewed, not as sources of error in QoL research but, rather, that these properties are intrinsic to all QoL measurement.

In this study, we used an individualised measure of quality of life, the SEIQoL-DW, as we felt that, by focusing on the unique choices of patients, we would be in a position to detect more clearly any response shifts that might occur. SEIQoL index scores did not reveal a significant improvement in IQoL 3 months after receiving high quality conventional dentures. However, when the baseline scores were derived based on the then-test, and when comparing then-test and post-test estimates, a significant improvement was seen. Response shifts had occurred in that patients had changed their criteria for assessing their quality of life from baseline to 3 months. It was only when this change was factored into the analysis that the improvement following treatment could be seen. The changes in the SEIQoL were highly complex but it is possible to gain some insight into their nature by looking at the various components of the measure i.e. cues, weights and levels.

Four out of every 5 patients (81%) nominated at least one different QoL cue at 3 months compared to baseline. Therefore, the elements that they considered most important for their quality of life changed over the study period. This represents a form of re-conceptualisation, one with which clinicians will be familiar. Patients change and adapt with time and in response to changing circumstances. The domains that might have been important for one's QoL before treatment may not be as important on a subsequent occasion. The same phenomenon can be seen with disease progression. Some patients with severe chronic conditions report higher QoL than do healthy individuals [15]. Significantly disabled or terminally ill patients sometimes report QoL similar to or higher than that of healthy controls [16]. One limitation of the SEIQoL-DW in this context is that, the respondent is only allowed to select 5 cues. If she chooses different cues on a subsequent occasion from those chosen previously, it could be argued (as we have done) that she has re-conceptualised what QoL means to her. But if she were allowed select as many cues as she wished and she included all of the cues previously chosen as well as any new ones, then this would be more likely to indicate re-prioritisation rather than re-conceptualization. Patients may also have used different words at each evaluation to refer to essentially the same area. This can be controlled by collecting detailed descriptions of the life areas chosen as well as including questions assessing patients' own perception of change.

Patients were asked at 3 months to indicate retrospectively their level of functioning on each of the cues chosen at baseline. In general, patients retrospectively rated their level of functioning on most of the cues as lower that they had done at the time. If we assume that they completed both assessments at 3 months using a single internal frame of reference, it seems reasonable to label this as re-calibration. It may be that the superior function associated with the quality dentures provided caused patients retrospectively to perceive their pre-treatment levels as worse on reflection.

Because the SEIQoL-DW weights are individualised, it is possible to measure changes in the relative importance of cues over time. We found that on average some weights (comparing pre-test and then-test weights) changed indicating that reprioritisation can occur. However, when comparing then-test and post-test weights we found no changes. This might be a true finding, or maybe patients simply applied the same weights they were using at T2 to the cues at T1. This may also partly be an artefact of the SEIQoL-DW procedure as the weights of all five cues selected by respondents are constrained to add to 100. Therefore, if the relative importance of one cue increases, the relative importance of at least one of the other four cues must diminish.

One of the major challenges in interpreting the results of this study is that 81% of the patients chose at least one different cue at 3 months compared to baseline. All patients were asked, in the then-test, to re-evaluate their baseline cues, whether they were the same or not. It seems likely that this process is different for those who chose the same and different cues at 3 months and this is worthy of further research. The sample size of 19% of patients who chose exactly the same cues at 3 months was too small to draw firm conclusions about the nature of response shift in this group.

Some studies have found that memory can influence the findings from the then-test [17]. A limitation with our study is that we did not control for recall bias and we did not compare the changes with any criterion measure of change [18,19]. However, receiving dentures is a significant and salient event and it seems likely that the influence of recall bias is minimal especially given the number of judgements a patient had to make and the 3 month gap between assessments. One alternative explanations for our findings of a discrepancy between prospective and retrospective assessments is that subjects may have expected that receiving high quality dentures should improve their health, an they retrospectively rated their initial health as lower to reflect this expectation, a cognitive mechanism known as the implicit theory of change [20]. Our interpretation of the results is based on the assumption that the retrospective then-test data provides a more valid indication of baseline IQoL for comparison with 3 month data than does the baseline assessment itself. If, however we assume that the retrospective judgement is biased and that the concurrent baseline assessment is more valid, our results would be interpreted differently and there would be no treatment effect. To support the response shift theory, we would need to show that the new information available to patients after receiving their dentures led to more valid judgments of their baseline scores. However, it is as yet unclear how one would determine which theory is more valid for a particular situation. It would be important to distinguish patients who's situation had improved or deteriorated from those who had changed their mind about what it means to have the best or worst possible outcome.

Recently, Schwartz and Rapkin have proposed a new psychometric model, which posits that the "true" PRO score is contingent on aspects of the appraisal process [14]. The appraisal of a construct like QoL may be related to culture, personality and situation and may vary across persons and over time [21-24]. Building on the response shift model, Schwartz and Rapkin have proposed using an Appraisal Profile [21]. They suggest that "rather than simply asking people to re-rate their baseline status using "today's criteria", we assess their appraisal processes to make those criteria explicit at each time in order to help characterise qualitative change". Improved knowledge about the ways in which patients appraise QoL might lead to more valid, reliable and responsive measures. Future studies need to disentangle the differing ways individuals appraise QoL and researchers must acknowledge the dynamic nature of QoL by empirically testing for response shift phenomena.

## Conclusion

Improvements in the IQoL in edentulous patients, following treatment with high quality dentures, were apparent only when patient adaptation over time, was taken into account. This study demonstrated that an IQoL measure such as the SEIQoL-DW can be used to assess re-conceptualisation and reprioritisation and can be applied as a then-test to control for recalibration.

## List of abbreviations

QoL quality of life

IQoL individualised quality of life

SEIQoL-DW Schedule for the Evaluation of Individualised Quality of Life – Direct Weighting

PRO patient reported outcomes

## Authors' contributions

CAO, DH and FH developed the core idea. CAO, DH and FH designed the study. LR and SH monitored the study. CAO, LR and SH conducted the literature search. SH and LR performed the statistical analyses. LR wrote the first draft of the paper. All authors critically reviewed and contributed to the final draft of the paper and all are guarantors.
